# Evaluation of oral citrulline administration as a mitigation strategy for fescue toxicosis in sheep

**DOI:** 10.1093/tas/txaa197

**Published:** 2020-10-30

**Authors:** Maslyn A Greene, James L Klotz, Jack P Goodman, John B May, Brittany E Harlow, William S Baldwin, James R Strickland, Jessica L Britt, F Neal Schrick, Susan K Duckett

**Affiliations:** 1 Department of Animal and Veterinary Sciences, Clemson University, Clemson, SC; 2 USDA-ARS, Forage Production Research Unit, Lexington, KY; 3 Department of Plant and Soil Sciences, University of Kentucky, Lexington, KY; 4 Department of Biological Sciences, Clemson University, Clemson, SC; 5 Department of Animal Science, University of Tennessee, Knoxville, TN

**Keywords:** amino acids, citrulline, fescue toxicosis, sheep

## Abstract

Gestating ewes consuming ergot alkaloids, from endophyte-infected (E+) tall fescue seed, suffer from intrauterine growth restriction and produce smaller lambs. Arginine (Arg) supplementation has been shown to increase birth weight and oral citrulline (Cit) administration is reported to increase arginine concentrations. Two experiments were conducted to: 1) evaluate if oral supplementation with Cit or water, to ewes consuming E+ fescue seed, increases lamb birth weight and 2) determine the effectiveness of Cit and citrulline:malate as an oral drench and elevating circulating levels of Cit to determine levels and dose frequency. In experiment 1, gestating Suffolk ewes (*n* = 10) were assigned to one of two treatments [oral drench of citrulline–malate 2:1 (CITM; 81 mg/kg/d of citrulline) or water (TOX)] to start on d 86 of gestation and continued until parturition. Ewes on CITM treatment had decreased (*P* < 0.05) plasma Arg and Cit concentrations during gestation. At birth, lambs from CITM ewes had reduced (*P* < 0.05) crude fat and total fat but did not differ (*P* > 0.05) in birth weight from lambs born to TOX ewes. In experiment 2, nonpregnant Suffolk ewes (*n* = 3) were assigned to either oral citrulline (CIT; 81 mg/kg/d), citrulline–malate 2:1 (CITM; 81 mg/kg/d of citrulline), or water (CON) drench in a Latin Square design for a treatment period of 4 d with a washout period of 3 d. On d 4, blood samples were collected at 0, 0.5, 1, 2, 3, 4, 6, 8, 10, 12, and 18 h post drench. Oral drenching of CIT and CITM increased (*P* < 0.0001) Cit concentrations within 2 h and levels remained elevated for 6 h. Apparent half-life of elimination for CIT and CITM were 8.484 and 10.392 h, respectively. Our results show that lamb birth weight was not altered with a single oral drench of citrulline–malate; however, lamb body composition was altered. The level and frequency of citrulline dosing may need to be greater in order to observe consistent elevation of Cit/Arg concentrations to determine its effectiveness in mitigating fescue toxicosis.

## INTRODUCTION

Administration of arginine (Arg) to rats with induced hypertension has been found to increase vasodilation response when compared to hypertensive untreated rats (Kitazono et al., 1996). Wu et al. (2016) reviewed the effects of Arg administration on the dam and developing fetus and noted the importance of arginine to the developing embryo as well as the relative safety of large dose supplementation. Intravenous arginine administration has also been show to increase systemic vasodilation (Lorente et al., 1993) and lamb birth weight (Lassala et al., 2010). Extracellular Arg is essential for the formation of nitric oxide, a vasorelaxant, in mesenteric artery tissue (MacKenzie and Wadsworth, 2003), and increasing circulating Arg concentrations induces vasodilation in humans and sheep (Lorente et al., 1993; Bode-Boger et al., 1998). In rats, blocking the production of nitric oxide by inhibiting nitric oxide synthase causes symptoms of preeclampsia and intrauterine growth restriction (IUGR; Yallampalli and Garfield, 1993). Unfortunately, Arg is almost completely degraded by rumen bacteria (Chalupa, 1976). Citrulline (Cit) may be a viable alternative to arginine, as it is part of the urea cycle and can be converted into Arg (Ah Mew et al., 2003). Citrulline has been confirmed to survive rumen degradation in vitro and in vivo with sheep (Gilbreath et al., 2020a) and cattle (Gilbreath et al., 2020b). Additionally, Cit has been shown to be more effective at elevating Arg concentrations when administered intravenously (Lassala et al., 2009).

Tall fescue [*Lolium arundinaceum* (Schreb.) Darbysh], a common cool-season perennial forage in the southeastern United States, is commonly infected with an endophytic fungus (*Epichloe coenophiala*; previously *Neotyphodium coenophialum*) (Ball et al., 2007). The fungus and plant have a symbiotic relationship that allows the plant to have an overall hardiness, which is desirable compared to other forage species in the area. Unfortunately, the fungus also produces secondary metabolites known as ergot alkaloids (Stuedemann and Hoveland, 1988), which have been linked to a condition known as fescue toxicosis. This condition is characterized by decreased weight gain (Schmidt et al., 1982), reproductive problems (Peters et al., 1992; Porter and Thompson, 1992), and vasoconstriction (Dyer, 1993; Klotz et al., 2010; Aiken and Flythe, 2014). Feeding endophyte-infected (E+) fescue to pregnant livestock has been reported to cause IUGR that is evident at d 133 of gestation in sheep (Greene et al., 2019) and at birth in sheep and cattle (Watson et al., 2004; Duckett et al., 2014a). Reductions in fetal weight caused by ergot alkaloid exposure are speculated to be due to placental insufficiency caused by increased vasoconstriction (Britt et al., 2019). Additionally, Klotz et al. (2019) found that ovine dams dosed with ergot alkaloids from tall fescue seed had increased vasoconstriction in umbilical arteries. Limited research has been conducted to determine if the oral administration of citrulline may improve lamb birth weight or IUGR associated with dam consumption of ergot alkaloids. The two objectives of this study were to: 1) evaluate if oral supplementation with Cit or water, to ewes consuming E+ fescue seed, increases lamb birth weight and 2) determine the effectiveness of citrulline and citrulline:malate as an oral drench in elevating circulating levels of Cit to determine dosing levels and frequency of dose.

## MATERIALS AND METHODS

All animal and experimental protocols were approved prior to start by the Clemson University Institutional Animal Care and Use Committee (AUP2017-061 and AUP2018-050). All live animal research was conducted at the Clemson University Small Ruminant Facility.

### Experiment 1

#### Experimental design.

Suffolk ewes were obtained from the Clemson University Small Ruminant Facility’s flock. Prior to breeding, ewes (*n* = 22, 86 ± 24 kg) were weighed and assigned a body condition score (1–5; with 5 = heaviest condition; 1 = thinnest condition; Sheep Prod. Industry Dev. Program, 2016). Ewes were divided into two groups (*n* = 11/group) for estrus synchronization and to facilitate breeding to a single ram. Over a 2-w interval during November of the fall breeding season, each group was synchronized using an intravaginal controlled internal drug release (CIDR) insert (Eazi-Breed CIDR, Zoetis Animal Health) for a period of 7 d. After 7 d, the CIDR was removed and an injection of prostaglandin F_2α_ (12.5 mg i.m.; Lutalyse; Pfizer, New York, NY) was administered (Merck Veterinary Manual, 2014). Ewes were then turned in with a purebred Suffolk ram fitted with a marking harness. The ewes were checked twice daily for crayon marks to estimate breeding date. Ewes were exposed to the ram for a 17-d period. Pregnancy was confirmed by abdominal ultrasonography (BCF Easi-Scan Curve Ultrasound, BCF Ultrasound, Rochester, MN) on approximately d 55 of gestation. Ewes that were confirmed pregnant (*n* = 13, 87 ± 25 kg) were also evaluated for twin and singleton pregnancies and randomly assigned to one of two treatment groups, E+ tall fescue seed with an oral drench of citrulline–malate 2:1 (CITM) or with a water drench (TOX). Low pregnancy rate is attributed to issues with ram fertility and short exposure. All ewes were marked and prior to the ram being removed.

Ewes were paired across treatments based on estimated breeding date to accurately assess gestational age for the start of treatment. All ewes were individually fed E+ tall fescue seed with either citrulline:malate oral drench treatment (CITM, *n* = 6) or with water only (TOX, *n* = 7). Treatment began on d 86 of gestation and continued until parturition. This time period (d 86 to parturition) was chosen based on previous research that identified d 86 to d 133 as a critical time for fetal growth when exposed to E+ tall fescue seed (Britt et al., 2019; Greene et al., 2019). Two ewes, H_2_O, were removed from the study after the start of treatment due to abortions, and one CITM ewe was removed due to having a singleton female. The final treatment groups were CITM (*n* = 5, three twin pregnancies, two singleton pregnancies) and TOX (*n* = 5, three twin pregnancies, two singleton pregnancies).

Defender Turf-type E+ tall fescue seed was used for the experiment and was grown in Oregon and acquired from Lewis Seed Warehouse (Louisville, KY). Seed samples were analyzed for ergot alkaloid content based on the protocol established by Aiken et al. (2009). Ergovaline and ergovalinine concentrations in Defender seed were measured at 3.76 μg/g dry matter (DM). Ergotamine was not detected in the seed samples. Seed was fed to ewes daily in a total mixed ration (TMR) at a total dose level to supply 1772 μg hd^−1^ d^−1^ of ergovaline and ergovalinine and was based on previous research (Aiken et al., 2009; Duckett et al., 2014a; Britt et al., 2019). Seed and TMR samples were taken for nutrient analysis, and daily rations were established to meet NRC requirements for gestating ewes from d 86 to parturition (Britt et al., 2019; Table 1). Seed was mixed by hand with TMR daily and fed to each ewe individually. Ewes in both treatments received equal amount of seed and TMR in order to minimize feed intake difference across treatments. Ewes were individually fed at 06:45 a.m. and given 2 h to eat. Orts were recorded for each ewe to calculate actual dry matter intake (DMI).

**Table 1.** Nutrient composition of diet, total mixed ration (TMR), endophyte-infected tall fescue seed, and citrulline:malate drench

TMR composition	% of ration, DM basis	
Corn grain, cracked	35.0	
Cottonseed hulls	25.0	
Soybean hulls	20.5	
Molasses	14.0	
Soybean meal	4.5	
Limestone	1.0	
**Tall fescue seed**	**kg hd** ^**−1**^ **d**^**−1**^ **DM**	**Ergovaline + Ergovalinine content, mg hd** ^**−1**^ **d**^**−1**^ **DM**
Endophyte-infected (E+) seed, tall fescue defender turf type	0.44	1.77
**Total nutrient composition**	**kg hd** ^**−1**^ **d**^**−1**^ **DM**	
LATE gestation, d 86 – parturition		
TDN	1.42	
Crude protein	0.22	

Citrulline–malate 2:1 was acquired from BulkSupplements.com (Henderson, NV). Citrulline–malate 2:1 was tested for purity via high-performance liquid chromatography (HPLC), as described below, and was 99.9%. Dose level administered to each CITM ewe (*n* = 5) was 81 mg/kg of citrulline per day. This dose was determined based on previous studies using arginine supplementation of pregnant sheep (Lassala et al., 2009; Lassala et al., 2010, 2011; Satterfield et al., 2013; Wu et al., 2016). Average ewe body weight of CITM ewes at d 85 of gestation prior to the start of treatment was 90 ± 7 kg. All CITM ewes received the same dose of 7.29 g of citrulline from d 86 of gestation to parturition, which provided less than 5% of their daily nitrogen intake. This dose Citrulline was chosen over arginine due to the extremely high degradation of arginine by rumen bacteria (Chalupa, 1976; Gilbreath et al., 2020b). Citrulline dose was delivered as an individual, oral drench (10.935 g citrulline-malate 2:1/35 mL ultrapure water) prior to feeding each morning. Citrulline:malate 2:1 was chosen rather than pure Cit because it dissolved into a solution with a significantly smaller volume of water. TOX ewes (*n* = 5) were given a drench of ultrapure water of equal volume prior to feeding each morning.

#### Maternal sample collection.

 Blood samples were collected from ewes on d 85, 99, 113, 120, 127, 130, 134, and 140. Fasted blood samples were collected prior to morning drenching and feeding. Whole blood samples were collected by jugular venipuncture into serum and EDTA-coated collection tubes. Plasma samples were immediately placed on ice following collection and plasma was separated by centrifugation at 537 × *g* for 20 min at 4 °C. Plasma samples were stored at −20 °C for subsequent analysis. Whole blood was allowed to clot for 30 min at room temperature, stored at 4 °C for 24 h, then serum was separated by centrifugation at 537 × *g* for 20 min at 4 °C. Serum was stored at −20 °C for future analysis.

#### Doppler ultrasound.

 Doppler ultrasound was used to examine blood flow in the carotid artery of all ewes on d 76 ± 4 of gestation, prior to treatment, and d 112 ± 4 of gestation, 27 ± 4 d after the start of treatment. Color Doppler ultrasound images of left carotid artery cross-sections were collected using a Classic Medical TeraVet 3,000 Ultrasound Unit (Classic Universal Ultrasound, Tequesta, FL) with a 12L5-VET (12 MHz) linear array transducer. Each imaging session was started at approximately 0900 hours and was completed within 1 h. The ewes were accustomed to regular handling and being in their individual feeding pens prior to the start of the study to reduce excitability when being imaged. Ewes were placed in individual feeding pens for imaging, with each individual imagining session taking no more than 5 min. Each ewe was clipped 1 d prior to scanning at the ultrasound site prior to each imaging session using surgical clippers. Scan depth was set at 4 cm. Cross-sectional images were collected for each artery using a frequency of 5.0 MHz and a pulse repetitive frequency that ranged between 2.5 and 3.0 kHz. Following freezing of an individual scan, frames stored in the cine memory of the unit were searched to store the image exhibiting the maximum flow signal, assumed to be at peak systolic phase. The flow signal was traced to estimate lumen area (Aiken et al., 2009). Results are expressed as the difference between the pretreatment (d 76 ± 4) and post endophyte-infected tall fescue treatment (d 112 ± 4) measures.

#### Lamb harvest and sample collection.

 Within 12 h of birth, birth weight, and crown to rump length were measured for all lambs. Then the first-born male lamb was harvested (*n* = 5/treatment). The attending veterinarian euthanized the lamb with an intravenous overdose of pentobarbital. Following euthanasia, the hide, head, feet, and tail were removed and the carcass was eviscerated and a weight of the carcass was obtained. From the viscera and carcass, weights of the fat masses (kidney fat, heart fat, and scapular fat) were collected. Samples of the brain, heart, kidney, and liver were taken and stored at −20 °C for later fatty acid analysis. Scapular and heart fat were snap frozen in liquid nitrogen and stored at −80 °C for RNA extraction. An aliquot of liver tissue was snap frozen in liquid nitrogen and stored at −80 °C for western blot analysis. From the right side of each lamb, all fat and muscle were removed for total body proximate composition analysis and fatty acid analysis.

#### Proximate composition.

 From the right side of each lamb, total muscle and fat samples were chopped and homogenized (Blixer 3 Series D, Robot Coupe Inc., Ridgeland, MS). Afterward, an aliquot was removed for moisture content analysis. The remaining sample was frozen at −20 °C, lyophilized (VirTis, SP Scientific, Warminster, PA), ground (Blixer 3), and stored at −20 °C. Samples were analyzed in duplicate for nitrogen content by the combustion method utilizing a Leco FP-2000 nitrogen analyzer (Leco Corp., St. Joseph, MI). Nitrogen content was multiplied by 6.25 to determine the crude protein content of the sample. Moisture content was determined by weight loss after drying samples for 24 h at 100 °C. Ash content was determined by ashing samples for 8 h at 600 °C.

#### Fatty acid analysis.

 Fatty acid analysis was conducted on the brain, liver, kidney, heart, and total lean tissue collected from the right side of each lamb and follows the methods previously established by Duckett et al. (2014a). Tissue samples were freeze dried and transmethylated following the protocol of Park and Goins (1994). Fatty acid methyl esters (FAME) were examined using a gas chromatograph (Agilent 6850, Agilent, San Fernando, CA) in combination with an automatic sampler (Agilent 7673A, Hewlett-Packard, San Fernando, CA). Separations were achieved with a 120-m TR-FAME (Thermo Fisher, Greenville, SC) capillary column (0.25 mm i.d. and 0.20 μm film thickness). Column oven temperature rose from 150 to 160 °C at a rate of 1 °C/min, then from 160 to 167 °C at a rate of 0.2 °C/min, and then from 167 to 225 °C at a rate of 1.5 °C/min. Once 225 °C was reached, the column temperature was maintained for 16 min. Both the injector and flame-ionization detector were maintained at 250 °C. Samples were injected at a volume of 1 μL. Hydrogen was used as the carrier gas, with a flow rate of 1 mL/min. Each sample was run twice for analysis, first with a split ratio of 100:1 for trans C18:1 and long chain-fatty acids. Then again, with a split ratio of 10:1 for omega-3 fatty acids and conjugated linoleic acid. Fatty acids were identified by evaluating the retention times with known standards (Sigma, St. Louis, MO; Supelco, Bellefonte, PA; Matreya, Pleasant Gap, PA) Measurement of fatty acids in each sample was accomplished by adding an internal standard, methyl tricosanoic (C23:0), during methylation, and expressed as a percentage of the total fatty acid weight. Total lipid content was analyzed using an Ankom XT-15 Extractor (Ankom Technology, Macedon, NY), with hexane as a solvent and samples were run in duplicate.

#### Blood analyte analysis.

 Serum prolactin levels were measured throughout late gestation by radioimmunoassay using the procedures of Bernard et al. (1993). Average intra-assay variance was 4.71% and inter-assay variance was 5.75%. Plasma glucose levels were evaluated with colorimetric assay using a hexokinase reagent (Pointe Scientific, Canton MI). Average intra-assay variance was 9.19% and inter-assay variance was 8.45%. Plasma insulin was measured using a Mercodia Ovine Insulin Kit (Mercodia, US, NC). Average intra-assay variance was 7.59% and inter-assay variance was 4.57%. Plasma glucose and insulin assays were validated for use in ovine samples previously (Duckett et al., 2014b; Long et al., 2014; Volpi-Lagreca and Duckett, 2017). Serum nonesterified fatty acids (NEFA) were quantified using a MaxDiscovery NEFA Assay (Bioo Scientific, Austin, TX). Average intra assay variance was 7.37% and inter-assay variance was 10.48%. Serum NEFA enzyme-linked immunosorbent assay (ELISA) kits were validated by comparison to chloroform–methanol extraction and GLC analyses of nonesterified, free fatty acids. Insulin sensitivity, RQUICKI, was calculated according to Holtenius and Holtenius (2007), using glucose, insulin, and NEFA concentrations.

Plasma amino acid analysis was conducted at the USDA-ARS, Forage Production Research Unit, Lexington, KY. In brief, amino acid standards were acquired from VWR. Plasma samples (250 µL) were spiked with internal standard solution (20 µL of 100 µM/µL sample of α-amino butyric acid) and proteins precipitated with the addition of cold methanol followed by centrifugation. The resulting solutions containing serum amino acids were derivatized with 6-aminoquinolyl-*N*-hydroxysuccinimidyl carbamate, AQC (Waters AccQ-Tag; Waters Corp, Milford, MA). Analyte concentrations were determined by liquid chromatography tandem mass spectrometry on a Waters Acquity tandem quadrupole detector (TQD) equipped with a Waters Acquity Sample Manager and Binary Solvent Manager. Chromatographic separation was obtained using a reverse-phase ultra-performance liquid chromatography (UPLC) column (Waters BEH C18, 1.7 μm, 2.1 mm × 150 mm). Column temperature was 40 °C. The mobile phase employed a mixture of water containing 0.1% formic acid (solvent A; Fisher) and acetonitrile (Fisher Optima) containing 0.1% formic acid (solvent B) in a linear gradient from 5% B to 75% B at a flow rate of 0.35 mL/min. The TQD tandem mass spectrometer was operated in positive ion multiple reaction monitoring (MRM) mode with electrospray ionization using nitrogen as the nebulizing gas and argon as the collision gas. Quantitation was done using QuanLynx software, linear calibration curve, internal standard method.

#### Real-time PCR.

 Total ribonucleic acid (RNA) was extracted from scapular and heart fat samples (100 mg of tissue) using Trizol reagent (Invitrogen, Thermo Fisher Scientific, Waltham, MA) in accordance with the manufacture specifications. Samples were cleaned of genomic deoxyribonucleic acid (DNA) using a DNA-free Kit (Ambion, Carlsbad, CA) according to the manufacturer specifications. The RNA yield was measured using a NanoDrop 1 spectrophotometer (Thermo Scientific, Thermo Fisher, Waltham, MA). Extracted RNA was stored at −80 °C after isolation. Samples were checked for quality after DNase treatment using an Agilent 2100 Bioanalyzer (Agilent Technologies, Santa Clara, CA). Two micrograms of RNA, along with qScript complementary DNA (cDNA) SuperMix (Quanta Bio, Beverly, MA), was used for cDNA synthesis according to the manufacturer instructions. Following conversion, cDNA was stored at −20 °C.

Perfecta (Quanta Bio, Beverly, MA) SYBR green and an Eppendorf Realplex Mastercycler (Eppendorf AG, Hamburg, Germany) were used, according to manufacturer instructions, for quantitative reverse transcriptase-polymerase chain reaction (qRT-PCR) analysis of gene expression. The PCR program used was a preliminary hold at 95 °C for 2 min immediately followed by 40 cycles of 95 °C for 15 s and 60°C for 30 s. Primers were designed to span exon boundaries using Primer 3 software (Table 2). Uncoupling protein 1 (UPC1; Symonds, 2013) and myogenic factor 5 (MYF5; Seale et al., 2008) were used to confirm the presence of brown adipose tissue (BAT). Fatty acid binding protein 4 (FABP4) was used to assess the presence of differentiated adipocytes (Shan et al., 2013). Glyceraldehyde 3-phosphate dehydrogenase (GAPDH), β-actin (ACTB), and cyclophilin B protein (CYC) were tested for stability using RefFinder (Xie et al., 2012) and the most stable housekeeping gene (CYC) was selected for normalization. Gene expression is displayed as the relative abundance from the control (TOX).

**Table 2.** Primer sequences used for RT-PCR analysis of lamb fat tissues

Gene	Forward, 5′-3′	Reverse, 5′-3′	Efficiency
UCP1	TGGGGATCTTTGCTAACCAG	ATGTTTTGCTTCCCCTTCCT	0.91
MYF5	GATTCTCAGCCTGCAACTCC	ATTTTTGGTGCCTCCTTCCT	1.03
FABP4	CATCTTGCTGAAAGCTGCAC	AGCCACTTTCCTGGTAGCAA	1.00
CYC	GGTCATCGGTCTCTTTGGAA	TCCATCACACGATGGAA	1.01
ACTB	GGGCAGTGATCTCTTTCTGC	CTCTTCCAGCCTTCCTTCCT	1.03
GAPDH	GGGTCATCATCTCTGCACCT	GGTCATAAGTCCCTCCACGA	1.01

#### Western blots.

 A subset of lambs (*n* = 4/treatment) was selected for analysis of cytochrome P450 subfamily 3A (CYP3A) activity using 30 µg microsomal protein from neonatal livers according to the procedures of Kumar et al. (2017). Due to the ability to recognize several proteins, CYP3A subfamily was assessed rather than individual proteins (Acevedo et al., 2005; Damiri et al., 2012). In brief, 10% polyacrylamide gel was used to separate proteins by gel electrophoresis (SDS-PAGE) and transferred to a 0.45 µm nitrocellulose (Bio-Rad). Molecular weight markers were a pre-stained protein standard (Bio-Rad). Wild-type mouse liver, 20 µg, was used as a positive control for antibody reactivity. The blot was blocked using 1% skim milk/0.1% Tween 20 suspended in phosphate buffered saline. Equal loading of samples was accomplished using rabbit anti-mouse β-actin (Sigma Aldrich, St. Louis, MO). The primary antibody was rabbit antirat CYP3A1 (Chemicon International, Temecula, CA) diluted 1:1000. Secondary antibody used for recognition of CYP3A subfamily was goat anti-rabbit IgG (Bio-Rad) alkaline-phosphatase diluted 1:500. A chemiluminescent kit (Bio-Rad) was used for visualization of the bands according to the manufacturer recommendations. Quantification of chemiluminescence was done using a Chemi-Doc system coupled with Image Lab software (Bio-Rad).

#### Statistical analysis.

 The univariate procedure of SAS (SAS, version 9.4) was used to test all variables for normality. Serum prolactin concentrations were not normally distributed (Shapiro-Wilk test *P* < 0.01) and therefore, were log-transformed to preserve normality. Data were analyzed using the mixed procedure of SAS with citrulline treatment (CITM vs. TOX) in the model. Ewe (lamb) was the experimental unit for all analyses. For ewe plasma and serum analytes, repeated measures analyses with autoregressive covariance structure was used to evaluate treatment, day of gestation and the two-way interaction in the model and the Kenward–Roger method was used to generate degrees of freedom. Lamb number (single or twin) was included as a covariate when significant (*P* < 0.05). Least square means were generated and separated using a protected least significant difference test. Statistical significance was determined at *P* < 0.05 with trends at *P* < 0.10.

### Experiment 2

#### Experimental design.

 A 3 × 3 Latin Square design was used to determine the effects of oral citrulline and citrulline–malate 2:1 on circulating citrulline concentrations of nonpregnant ewes without the addition of E+ fescue treatment. Suffolk ewes (*n* = 3, BW 42 ± 7 kg) were selected and administered either citrulline (CIT), citrulline:malate (CITM), or an equal volume of water (CON) as a drench immediately prior to feeding. Dose level administered to each CIT and CITM ewe consistent with experiment 1. Ewes were fed once daily and maintained on the same TMR diet as experiment 1 that was formulated to meet NRC requirements. The experimental period was 7 d, with 4 d of treatment drench and 3 d of washout. Blood samples were collected on the fourth day of treatment as a serial collection. Jugular cannulas were placed ~18 h prior to sampling and flushed every 4 h with sodium citrate until the start of sampling.

#### Blood sampling.

 Whole blood was collected from preplaced cannulas with a syringe immediately prior to drenching (0 h) and at time 0.5, 1, 2, 3, 4, 6, 8, 10, 12, and 18 h post drenching. Whole blood was allowed to clot for 30 min at room temperature and then stored for 24 h at 4 °C and then, serum was separated by centrifugation at 537 × *g* for 20 min at 4 °C. Serum was stored at −20 °C for future amino acid analysis. Serum amino acids were measured using the same procedures at experiment 1. Plasma and serum Cit concentrations were evaluated from experiment 1 and there was no difference (*P* = 0.74) between the levels of Cit in the plasma and serum samples.

#### Statistical analysis.

 Data were analyzed as a 3 × 3 Latin Square using a repeated measure with PROC GLMMIX (SAS, version 9.4). Terms in the model included ewe, period, collection time, and treatment. The Kenward–Roger method was used to generate degrees of freedom. Area under the curve (AUC) was calculated using the trapezoid method (Le Floch et al., 1990). The absorption and elimination coefficients (*k*_a_ and *k*_e_) for circulating citrulline were calculated using the serum citrulline concentrations according to Renwick (1994). The apparent half-life of elimination for citrulline was calculated using the formula *t*_1/2_ = ln 2/*k*_e_ according to Moinard et al. (2008). The AUC, *k*_a_, *k*_e_, and *t*_1/2_ were analyzed using PROC GLM and least square means were generated and separated using a protected least significant difference test. Statistical significance was determined at *P* < 0.05 with trends at *P* < 0.10.

## RESULTS

### Experiment 1: Maternal Characteristics

Ewe plasma amino acid concentrations by citrulline treatment are shown in Table 3. All interactions between citrulline treatment and day of gestation were non-significant (*P* > 0.10) for all blood hormone and metabolites analyzed. Drenching with citrulline–malate did not alter (*P* > 0.10) plasma glutamate (Glu), ornithine (Orn), valine (Val), leucine (Leu) or isoleucine (Ile) concentrations compared to water (TOX). Plasma Arg and Cit concentrations were reduced (*P* < 0.05) for CITM compared to TOX ewes. Additionally, CITM ewes had reduced (*P* < 0.05) phenylalanine (Phe) and lysine (Lys) concentrations while plasma Gln tended (*P* = 0.06) to be reduced compared to TOX ewes.

**Table 3.** Ewe plasma amino acid concentrations (µM) in ewes fed endophyte-infected tall fescue seed and drenched with citrulline-malate (CITM) or water (TOX) from d 86 to parturition (experiment 1). All interactions between citrulline-malate treatment and day of gestation were nonsignificant (*P* > 0.40) and results are presented as main effects

Treatment	CITM	TOX	SEM	*P*-Level
*n*	5	5		
Arginine	125.8	153.7	6.20	0.02
Citrulline	250.0	287.3	9.76	0.03
Glutamate	300.3	300.6	10.82	0.98
Glutamine	35.72	54.49	6.050	0.06
Isoleucine	115.2	121.1	5.75	0.49
Leucine	117.6	126.5	5.91	0.32
Lysine	84.70	100.87	4.065	0.03
Ornithine	56.61	59.04	2.846	0.57
Phenylalanine	59.77	74.08	2.900	0.01
Valine	130.2	146.8	7.49	0.16

Concentrations of serum prolactin, plasma glucose and insulin concentration, and glucose:insulin ratio were not altered (*P* > 0.10) by citrulline–malate treatment (Table 4). Concentrations of NEFA tended to be elevated (*P* = 0.06) for CITM ewes during late gestation. Revised Quantitative Insulin Sensitivity Check Index, RQUICKI, can be calculated based on concentrations of glucose, insulin, and NEFA in circulation (Holtenius and Holtenius, 2007). In this study, RQUICKI values did not differ (*P* = 0.17) by citrulline:malate treatment.

**Table 4.** Ewe glucose, insulin, nonesterified fatty acids (NEFA), and prolactin concentrations in ewes fed endophyte-infected tall fescue seed and drenched with citrulline–malate (CITM) or water (TOX) from d 86 to parturition (experiment 1)

Treatment	CITM	TOX	SEM	*P*-Level
*n*	5	5		
Prolactin, ng/mL	19.14	13.53	6.350	0.87
Glucose, mg/dL	57.43	61.28	1.797	0.17
Insulin, μg/L	0.557	0.610	0.159	0.81
Glucose:insulin	158.18	135.51	29.48	0.62
NEFA, mmol/L	0.337	0.242	0.035	0.06
RQUICKI^*a*^	1.07	1.52	0.221	0.17

All interactions between citrulline–malate treatment and day of gestation were nonsignificant (*P* > 0.40) and results are presented as main effects.

^*a*^RQUICKI calculated according to Holtenius and Holtenius (2007).

Ewe characteristics are presented in Table 5. Citrulline-malate treatment did not alter (*P* > 0.10) ewe body weight, average daily gain, dry matter intake (DMI), or gestation length (data not shown). Carotid artery luminal area was measured on d 112 of gestation, 36 d after the E+ fescue inclusion in the diet, and tended (*P* = 0.06) to be reduced when compared to pretreatment (d 76 of gestation) carotid artery luminal area regardless of Cit treatment (data not shown). Citrulline-malate drench did not impact (*P* = 0.37) carotid artery luminal area difference between CITM and TOX ewes. Additionally, the number of lambs born, lamb birth weight, and average crown rump length per ewe did not differ (*P* > 0.10) by citrulline treatment.

**Table 5.** Effect of dosing citrulline-malate (CITM) vs. water (TOX) to ewes exposed to endophyte-infected tall fescue on maternal characteristics in experiment 1

Treatment	CITM	TOX	SEM	*P*-Value
Ewe (*n*)	5	5		
Ewe BW on d 85, kg	88.85	83.89	4.844	0.49
Ewe BW on d 140, kg	109.64	105.10	4.452	0.49
Ewe ADG, kg/d	0.378	0.385	0.0195	0.79
Ewe DMI, kg/d	2.44	2.37	0.044	0.29
Ewe Doppler ultrasound^*a*^				
Pretreatment carotid artery area d 76, cm^2^	36.26	34.17	2.333	0.54
Treatment carotid artery area d 112, cm^2^	29.10	31.39	3.269	0.63
Carotid artery area difference, cm^2^	−7.16	−2.78	3.234	0.37
Lamb characteristics per ewe				
Lambs born/ewe	1.60	1.60	0.245	1.00
Birth weight, g/ewe	7936.1	7649.4	293.55	0.51
Avg. crown-rump, cm	52.33	52.00	0.344	0.52
Lamb carcass, g	2456.7	2526.5	226.12	0.83
Lamb fat depots, g				
Scapular fat	5.88	11.78	1.894	0.06
Kidney fat	31.20	43.70	4.227	0.07
Heart fat	8.85	4.38	2.186	0.20
Lamb fat depots, % of empty body weight				
Scapular fat	0.13	0.27	0.049	0.09
Kidney fat	0.68	0.93	0.058	0.02
Heart fat	0.20	0.09	0.055	0.20
Lamb body composition, g				
Total fat-free lean	1148.7	1142.2	92.38	0.96
Total bone	1307.9	1384.3	146.65	0.72
Total fat	71.50	100.01	7.285	0.02
Lamb proximate composition, %				
Moisture	77.72	77.02	0.425	0.28
Crude protein	16.33	16.11	0.276	0.58
Crude fat	2.29	3.20	0.207	0.01
Ash	0.97	0.98	0.026	0.83

^*a*^Doppler ultrasound measurements are the difference in luminal area of the carotid artery taken on d 112 after feeding endophyte-infected tall fescue seed for 26 d compared to pre-treatment (d 76) levels.

### Experiment 1: Lamb Characteristics

One male lamb (*n* = 10, five CITM, five TOX) was harvested from each ewe for body compositional analyses (Table 5). Lamb carcass weight did not differ (*P* > 0.10) by maternal citrulline-malate treatment. Total fat mass, and crude fat percentage were reduced (*P* < 0.03) in lambs born to CITM ewes compared to TOX. Scapular fat (BAT) and kidney fat weight tended to be reduced (*P* < 0.07) in lambs from CITM ewes. When normalized to an empty body weight, kidney fat and total body fat percentages were reduced (*P* < 0.05) for CITM compared to TOX lambs. Scapular fat mass also tended to be reduced (*P* = 0.09) in CITM lambs on an empty body weight basis. Scapular and heart fat both expressed UCP1 and FABP4 but did not differ in relative abundance between treatments (*P* > 0.10) or tissues (*P* > 0.10, data not shown). In both tissues, MYF5 was present in low abundance. Additionally, western blot analysis of lamb liver showed no difference (*P* = 0.17) for CYP3A presence in neonatal livers based on maternal citrulline–malate treatment (data not shown).

Fatty acids were analyzed by maternal treatment (CITM vs. TOX), tissue (brain, heart, liver, kidney, and total lean) and the interaction. The interaction between maternal treatment and tissue was non-significant (*P* > 0.05). Maternal citrulline–malate treatment increased (*P* = 0.02) eicosapentaenoic acid (C20:5 *cis*-5, 8, 11, 14, 17; EPA) in the tissues (Table 6). Eicosadienoic acid (C20:2, *cis*-11, 14) and dihomo-γ-linolenic acid (20:3 *cis*-8, 11,14) tended to be increased (*P* = 0.09, *P* = 0.05) for lambs from CITM ewes. Additionally, lambs from CITM ewes tended (*P* = 0.09) to have reduced saturated fat content.

**Table 6.** Experiment 1. Fatty acid profile of lambs from ewes consuming E+ fescue seed and drenched with citrulline:malate (CITM) or water (TOX)

Treatment	CITM	TOX	SEM	*P*-value
Fatty acid, %				
C14:0	1.59	1.61	0.327	0.97
C14:1 *cis*-9	0.13	0.24	0.109	0.51
C15:0	0.78	1.26	0.318	0.29
C16:0	21.25	22.05	0.438	0.21
C16:1 *cis*-9	2.37	2.53	0.088	0.21
C17:0	0.11	0.11	0.066	0.95
C18:0	14.14	14.93	0.422	0.20
C18:1 *trans*-10	0.38	0.27	0.089	0.41
C18:1 *cis*-9	35.54	35.76	0.544	0.79
C18:1 *cis*-11	3.89	3.82	0.117	0.67
C18:2 *cis*-9,12	0.93	0.82	0.082	0.34
C18:3 *cis*-9,12,15	0.040	0.040	0.0310	0.99
C20:0	0.19	0.20	0.015	0.51
C20:1 *cis*-11	0.63	0.64	0.019	0.83
C20:2 *cis*-11,14	0.127	0.098	0.0116	0.09
C20:3 *cis*-5, 8, 11	2.30	2.02	0.127	0.13
C20:3 *cis*-8,11,14	0.33	0.27	0.020	0.05
C20:4 *cis*-5,8,11,14	5.75	5.45	0.267	0.42
C20:5, *cis*-5,8,11,14,17	0.149	0.121	0.0082	0.02
C22:4 *cis*-7,10,13,16	1.28	1.35	0.034	0.12
C22:5, *cis*-7,10,13,16,19	0.62	0.57	0.036	0.35
C22:6, *cis*-4,7,10,13,16,19	2.91	2.78	0.129	0.47
Saturated	36.99	38.59	0.662	0.09
Odd-chain	0.80	1.29	0.319	0.29
Monounsaturated	38.10	38.47	0.561	0.65
Polyunsaturated *n−*6	1.39	1.20	0.09	0.12
Polyunsaturated *n−*3	3.66	3.45	0.154	0.36
Ratio, *n−*6:*n−*3	0.85	0.86	0.063	0.91

Simple effect of treatment displayed with means averaged from lamb total lean, liver, kidney, heart, and brain tissue,

When fatty acids were examined by tissue and not maternal treatment, 18 of the fatty acids analyzed were different at *P* < 0.01 (Table 7). Brain tissue had the largest concentration of pentadecyclic (C15:0), stearic (C18:0), *cis*-11 vaccenic (C18:1 *cis*-11), arachidic (C20:0), eicosenoic (C20:1), eicosaddienoic, docosatetraenoic (C22:4 *cis*-7,10,13,16), and docosahexaenoic (C22:6 *cis*-4,7,10,13,16,19; DHA); however brain tissue also had the least variety of different fatty acids, only 16, when compared to the other tissues. Brain tissue had the lowest concentration of oleic (C18:1 *cis*-9) and linoleic acids (C18:2 *cis*-9,12). Both the brain and the total lean had the lowest concentration of mead (C20:3 *cis*-5,8,11) acid. Mead acid is formed from oleic acid only when there is a deficiency in the essential fatty acids during development (Mead and Slaton, 1956; Mead, 1958; Ichi et al., 2014). Mead acid was present in lambs from both treatments indicating that maternal citrulline:malate treatment did not alter mead acid production. Brain tissue was primarily composed of saturated (42.97%), monounsaturated (19.55%), and polyunsaturated *n*−3 fatty acids (10.60%). Liver tissue had the most variety of fatty acids with 22 different species and was composed mainly of saturated (38.78%), monounsaturated (32.07%), and polyunsaturated *n*−3 fatty acids (3.62%). Additionally, myristoleic (C14:1), margaric (C17:0), and α-linolenic (C18:3 *cis*-9,12,15) acids were unique to liver tissue. In kidney, tissue monounsaturated (38.77%) and saturated fatty acids (34.48%) were the most abundant. Heart tissue contained mostly monounsaturated (47.20%) and saturated fatty acids (38.06%). The total lean contained the highest percentage of monounsaturated fatty acids (53.84%), while also containing the lowest concentrations of polyunsaturated *n*−6 and *n*−3 (0.68% and 0.56%). These results indicate that there are regional differences in the fatty acid composition of neonatal lamb tissues and that the brain contains the most *n*−3 fatty acids of the tissues examined.

**Table 7.** Experiment 1. Fatty acid profile of lamb total lean, liver, kidney, heart, and brain tissues

Tissue Type	Brain	Heart	Kidney	Liver	Total Lean	SEM	*P*-value
Fatty acid, %							
C14:0	1.00^f^	1.26^f^	1.53^f^	2.99^g^	1.25^f^	0.518	0.07
C14:1 *cis*-9	–	–	–	0.18	–	0.077	–
C15:0	2.65^a^	0.30^b^	0.58^b^	0.23^b^	1.36^ab^	0.504	0.01
C16:0	23.70^a^	20.25^b^	20.35^b^	23.91^a^	20.05^b^	0.692	<0.0001
C16:1 *cis*-9	–	1.83^a^	1.90^ac^	3.84^b^	2.24^c^	0.125	<0.0001
C17:0	–	–	–	0.11	–	0.047	–
C18:0	18.27^a^	16.55^a^	12.60^b^	11.88^b^	13.38^b^	0.668	<0.0001
C18:1 *trans*-10	–	–	–	0.53^a^	0.12^b^	0.089	0.01
C18:1 *cis*-9	18.10^a^	44.97^b^	36.41^c^	27.51^d^	51.26^e^	0.860	<0.0001
C18:1 *cis*-11	4.91^a^	3.40^bc^	3.65^b^	4.39^a^	2.91^c^	0.185	<0.0001
C18:2 *cis*-9,12	0.17^a^	1.25^b^	1.38^b^	1.05^b^	0.55^c^	0.130	<0.0001
C18:3 *cis*-9,12,15	*−*	*−*	*−*	0.040	*−*	0.022	*−*
C20:0	0.359^a^	0.170^bc^	0.222^b^	0.086^c^	0.133^c^	0.0233	<0.0001
C20:1 *cis*-11	1.45^a^	0.40^bd^	0.46^bc^	0.53^c^	0.33^d^	0.030	<0.0001
C20:2 *cis*-11,14	0.246^a^	0.038^b^	0.096^c^	0.153^d^	0.029^b^	0.0183	<0.0001
C20:3 *cis*-5, 8, 11	1.52^a^	1.58^a^	2.74^b^	3.44^c^	1.52^a^	0.201	<0.0001
C20:3 *cis*-8,11,14	0.429^a^	0.227^b^	0.372^a^	0.386^a^	0.099^c^	0.0311	<0.0001
C20:4 *cis*-5,8,11,14	7.98^a^	3.75^b^	8.24^a^	6.43^c^	1.59^d^	0.423	<0.0001
C20:5, *cis*-5,8,11,14,17	*−*	0.112^a^	0.221^b^	0.179^c^	0.028^d^	0.0116	<0.0001
C22:4 *cis*-7,10,13,16	4.12^a^	0.36^b^	0.54^c^	1.14^d^	0.41^bc^	0.054	<0.0001
C22:5, *cis*-7,10,13,16,19	0.51^a^	0.45^a^	0.58^a^	1.67^b^	0.28^c^	0.057	<0.0001
C22:6, *cis*-4,7,10,13,16,19	10.09^a^	0.52^b^	1.12^c^	2.23^d^	0.25^b^	0.203	<0.0001
Saturated	42.97^a^	38.06^b^	34.48^c^	38.78^b^	34.68^c^	1.046	<0.0001
Odd-chain	2.65^a^	0.30^b^	0.58^b^	0.34^b^	1.36^ab^	0.504	0.01
Monounsaturated	19.55^a^	47.20^b^	38.77^c^	32.07^d^	53.84^e^	0.888	<0.0001
Polyunsaturated *n−*6	0.84^a^	1.52^b^	1.84^b^	1.59^b^	0.68^a^	0.136	<0.0001
Polyunsaturated *n−*3	10.60^a^	1.07^b^	1.92^c^	3.62^d^	0.56^b^	0.244	<0.0001
Ratio, *n−*6:*n−*3	0.082^a^	1.470^b^	1.014^c^	0.477^d^	1.251^bc^	0.1000	<0.0001

Simple effect of tissue type displayed with means averaged across maternal treatments.

^abcde^Means in the same row differ *(P* < 0.05).

^fg^Means in the same row differ *(P* < 0.10).

### Experiment 2: Citrulline Concentrations

**Figure 1.**
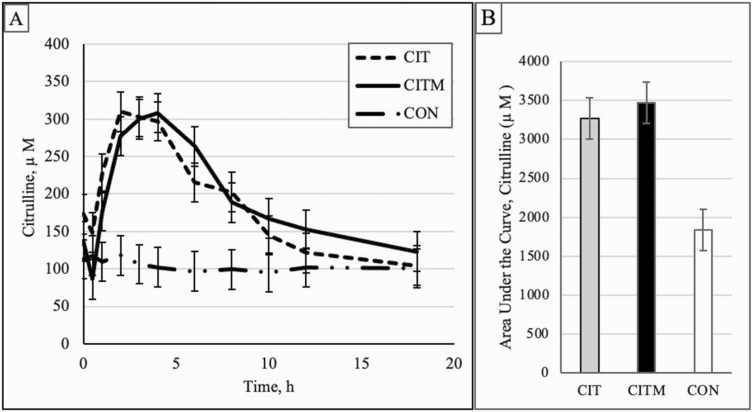
Citrulline concentrations (A) in ewe lambs drenched with an oral dose of citrulline (CIT), citrulline-malate (CITM), or water (CON; experiment 2). Area under the curve (B) for citrulline concentrations for CIT, CITM, and CON ewe lambs.

Oral drenching of citrulline and citrulline–malate 2:1 increased (*P* < 0.0001) citrulline levels during the 18 h sampling period (Figure 1). Citrulline concentrations were elevated between 1 and 8 h after oral drenching of CIT when compared to water drench (CON). Drenching CITM elevated circulating Cit levels between 2 and 8 h after drenching when compared to water. Citrulline treatment, both CIT and CITM, response did not differ from each other at any time point. Both CIT and CITM drench took 2 h to increase circulating Cit concentrations above levels at 0 h, and then levels remained elevated until 6 h post drenching. The AUC for Cit was increased (*P* = 0.01) for both CIT and CITM when compared to water (CON; Table 6). The apparent *k*_a_, *k*_e_, and *t*_1/2_ of Cit were estimated for each ewe and did not differ (*P* > 0.10) for CIT and CITM treatments. The average *k*_a_ for CIT was 0.8577 µM/h and the average *k*_e_ was 0.0817 µM/h. The average *k*_a_ for CITM was 1.2787 µM/h and the average *k*_e_ was 0.0667 µM/h. Apparent half-life of elimination for CIT and CITM were 8.730 and 12.073 h, respectively.

## DISCUSSION

The vasoconstrictive effects of ergot alkaloids are well documented in cattle (Foote et al., 2012; Aiken et al., 2013; Pesqueira et al., 2014) and sheep (Klotz et al., 2019). Approximately 21 d after exposure to ergot alkaloids induces fescue toxicosis, which is characterized by reductions in prolactin secretions, the urinary excretion of ergot alkaloids, and vasoconstrictive activity (Hill et al., 2000; Klotz et al., 2016). Experiment 1 ewes showed a reduction in carotid artery area when pre E+ exposure scans were compared to scans taken after E+ seed was incorporated into the diet. These results show that E+ fescue did cause vasoconstriction, however there was no difference between CITM and TOX ewes. Reductions in maternal weight gain and average daily gain have been reported in steers (Matthews et al., 2005) and lactating ewes fed E+ hay (Zbib et al., 2014) or gestating ewes fed E+ seed (Britt et al., 2019). Ewes consuming E+ fescue have been reported to have shorter gestation lengths when compared to ewes consuming nontoxic endophyte-free fescue (Duckett et al., 2014a; Britt et al., 2019). Additionally, exposure to ergot alkaloids from E+ fescue causes a reduction in prolactin levels by interacting with the D2-dopamine receptor (Strickland et al., 2011; Klotz, 2015; Britt et al., 2019). In this study, all ewes were fed E+ tall fescue seed and therefore differences in ewe measurements with E+ exposure may not have been observed without a negative control, or ergot alkaloid-free, treatment.

Citrulline:malate treatment had no effect on circulating glucose and insulin concentrations during gestation. Supplementing rumen protected arginine to pregnant ewes does not alter glucose concentrations in plasma during late gestation (Gootwine et al., 2020). Glucose is a primary energy source for the placenta and the fetus during gestation (Hay, 2006) and can be a major limiting nutrient for fetal development (Battaglia and Meschia, 1988). Citrulline:malate treatment tended to elevate NEFA concentrations for gestating ewes consuming E+ fescue seed. During gestation, NEFA levels will increase because the dam is mobilizing fat tissue to keep up with both fetal growth and metabolic need (Luther et al., 2007). The RQUICKI values did not differ based on citrulline:malate treatment. As gestation progresses, especially near term, insulin secretion and sensitivity decrease and can potentially contribute to cases of pregnancy toxemia (Duehlmeier et al., 2013).

The majority of fetal growth (80%) occurs during the last trimester in sheep (Rattray et al., 1974), for this reason we administered the citrulline drench during the late gestation (d 86 – parturition) time period in attempt to mitigate E+ fescue effects. Britt et al. (2019) reported feeding E+ tall fescue seed during late gestation (d 85 to d 133) reduced fetal weight per ewe by 15% compared to control. Additionally, fetuses from ewes consuming E+ fescue have reduced muscle weights when compared to fetuses from ewes consuming E− fescue (Duckett et al., 2014a; Greene et al., 2019). Supplementation with Arg has been shown to increase the number of live-born offspring and litter weight in ewes, pigs, and rats (Lassala et al., 2010, 2011; Wu et al., 2013). Lambs from CITM ewes did not show increased weight or size when compared to TOX lambs. Oral administration of citrulline:malate also reduced Arg and Cit concentrations in gestating ewes. These results are in contrast to previous reports with intravenous Cit administration where Cit was more effective at elevating circulating levels of Arg and Cit over 240 min period compared to Arg infusion in pregnant ewes (Lassala et al., 2009). No previous work has been reported on oral drench administration of Cit to gestating ewes, and samples from experiment 1 were collected 24 h after a single dose administration. Experiment 2 was conducted to evaluate how a single dose of Cit affects circulating Cit concentrations in sheep during the 24 h period and to provide evidence as to why the results from Experiment 1 were contrary to previous literature.

In experiment 2, Cit concentrations were increased in 2 h when an oral drench was used to administer treatment. Research by Gilbreath et al. (2020a) shows that rumen samples incubated with 5 mM of Cit showed no degradation of Cit after a 4 h period. In sheep, feeding 8 g of Cit results in a 117% increase in serum Cit concentrations over a 4 h period. In the second experiment, Cit levels increased by 149% in 2 h when compared to water. The increase in circulating Cit was 89% greater than baseline (0 h) levels. These results indicate that a dosing of citrulline:malate would need to be done every 6 h at this dose level to continuously maintain elevated citrulline levels in the blood. Human studies with Cit dosages have shown that there is a positive linear relationship between the half-life and Cit dose level (Moinard et al., 2008). Subjects administered an oral dose of 10 g of Cit dissolved in water had an apparent half-life was determined to be 1.01 h. Moinard et al. (2008) reported that Cit was at maximum concentration >1 h of oral administration and sharply declined thereafter. In the current study, maximum Cit concentrations were seen between 2 and 4 h for CIT and CITM treatments. The differences observed between the two studies could be related to differences in human and sheep digestive systems.

Scapular fat, kidney fat, and total body fat mass, and crude fat percentage of the proximate composition for lambs from CITM ewes were reduced when compared to lambs from TOX ewes. Duckett et al. (2014a) found that lambs at birth from ewes consuming E+ fescue during gestation had reduced fat masses when compared to lambs from ewes fed E− fescue. In contrast, Greene et al. (2019) did not report a difference in fat masses for fetuses on d 133 of gestation from ewes fed E+ or E− fescue seed. In the current study, lambs were allowed to be carried to term and this may have attributed to differences in fat mass accumulation as fetal lipid deposition occurs more near term (Symonds et al., 2016). In cases of IUGR, fetuses may be born with decreased fat deposition due to reduced nutrient transport to the fetus, however human studies have found IUGR fetuses have an increased incidence of obesity post maturity (Engelbregt et al., 2004; Fernandez-Twinn and Ozanne, 2006).

Fatty acid analysis of lamb tissues revealed that maternal citrulline:malate treatment is capable of altering fatty acid deposition. Duckett et al. (2014a) found that EPA concentrations of the total lean muscle were elevated for lambs from dams on E+ fescue when compared to E− lambs. Additionally, polyunsaturated *n−*6 fatty acids were found in larger concentrations for lambs from ewes consuming E+ fescue when compared to lambs from E− fescue. In experiment 1, lambs from CITM ewes had increased EPA concentrations and *n−*6 fatty acids, eicosadienoic and dihomo-γ-linoleic acid compared to lambs from TOX ewes. The eicosanoid (C20) family of fatty acids can be elongated and desaturated into downstream fatty acids such as DHA and DPA (Gurr et al., 2016). Additionally, eicosanoids can be biologically transformed into prostaglandins, thromboxanes, leukotrienes, and lipoxins. Maternal amino acid treatment may have altered the conversion of essential fatty acids to downstream long-chain *n−*3 and *n−*6 polyunsaturated fatty acids and/or other essential signaling molecules, warranting further research.

Individual tissue analysis showed differences in the fatty acid concentration of various tissue examined. Brain tissue had a 352% more DHA than liver tissue and had 3,889% more DHA than the total lean muscle tissue. Brain tissue is known to have a high concentration of DHA (Blank et al., 2002). The liver contained 318% more docosapentaenoic (C22:5 *cis*-4,7,10,13,16, DPA) than the total lean tissue, which is consistent with previous analysis of liver tissue compared to muscle in mature lambs (Malau-Aduli et al., 2016). α-Linolenic acid was only detected in liver samples in the current experiment. Malau-Aduli et al. (2016) showed that α-linolenic acid is present in adipose, heart, and liver tissue in mature lambs but was not found in muscle or kidney tissue. Additionally, oleic acid was most abundant in the muscle tissue of lambs from the current study. Previous work has shown that oleic acid is found in abundance in adipose tissue with muscle tissue being the second largest amount in mature sheep (Malau-Aduli et al., 2016).

The presence of BAT was examined by looking at the expression of biomarkers, UCP1 and MYF5 for lamb fat masses. Earlier reports have stated that lambs and calves are born with only BAT, which converts to white adipose tissue (WAT) within 3 weeks of birth (Thompson and Jenkinson, 1969; Gemmell et al., 1972; Casteilla et al., 1989). Lambs from CITM ewes showed no difference in the abundance of UCP1 or MYF5 when compared to lambs from TOX ewes. These findings are in contrast to Satterfield et al. (2013), who reported that arginine administration to gestating ewes increased perirenal BAT accumulation. All fat masses analyzed showed the presence of FABP4 and this indicates that the tissue analyzed is adipose (Shan et al., 2013). Both WAT and BAT are capable of expressing FABP4. Additionally, previous work in our lab has shown that UCP1 and MYF5 are present in abundance in scapular fat at d 133 of gestation, about 2 wk prior to parturition (Greene et al. 2019). Uncoupling protein 1 is used by BAT stores for thermogenesis and the loss or reduction of this ability may result in hyperthermia and death to the lamb (Clarke et al., 1996; Symonds, 2013). Brown adipose tissue originates from a myogenic line and expresses MYF5 as a cell marker (Seale et al., 2008). Expression of UCP1 in BAT peaks at or close to birth and drops significantly in the first 7 d postpartum and BAT is replaced with WAT (Pope et al., 2012). Little research has been done to explain how BAT is replaced by WAT. Mueller (2014) reported on the discovery of beige fat, a “browned” WAT, an adipose type that produces UPC1 but is from the WAT cell lineage and not the myogenic lineage so does not produce MYF5. The findings from the current study would indicate that the fat masses may be transitioning from BAT to WAT, due to the minimal expression of MYF5. Few studies have looked at the both UCP1 and MYF5 in fat masses from livestock animals, also few experiments have looked to describe the transition of BAT to WAT in adipose. Additional studies are needed to examine the cellular mechanisms and timeline for WAT replacement of BAT, and what, if any, role beige fat may play in the transition.

These experiments have demonstrated that the oral drenching of Cit and citrulline:malate can alter circulating Cit concentrations in nonpregnant ewes. Additionally, citrulline:malate supplementation to late gestation ewes consuming E+ fescue and can alter fetal deposition of fat and fatty acid profile; however, lamb birth weight was not affected when compared to lambs from TOX ewes when administered once daily. Citrulline:malate was more water soluble and determined to be equally effective at increasing circulating citrulline concentrations when compared to pure Cit. The dose level and frequency of Cit dosing may need to be more frequent, every 6 h, in order to observe consistent elevation of Cit concentrations over a 24 h period. Additional experiments are needed to determine if Cit, when maintained in an elevated steady state, is capable of mitigating fescue toxicosis in gestating ewes.
